# A New Discovery in the Service of Homœopathy

**Published:** 1881-11

**Authors:** Manuel Græter


### A NEW DISCOVERY IN THE SERVICE OF HOMŒOPATHY.

*Translated from "Populare Zeitschrift fur Homœopathie," Jan.* 1881,

BY PROF. MANUEL GRÆTER.

The second discovery, of which the readers of the last annual series of our journal have been partly informed, is NEURAL ANALYSIS.

The author of this is Professor Gustav Jæger, M. D. Like every new discovery, it is still an object of doubt in many cases, especially for those who think that the science of to-day has already reached an infinitely high point and placed secure termini where knowledge and perception come to a termination and faith or superstition, the supernatural, have to begin.

In addition, those who in Prof. Jæger's theory of the soul perceived a contradiction to the teachings of religion felt called upon to attack him. Now, Prof. Jæger, apparently without any justification, had employed the word "Soul" for quite other things—for odorous substances of different quality which are developed in our body, and which, partly within the latter, react upon our mental disposition, our mind, and influence it in the most different manner, partly, also, evolving themselves outwardly; and, though often not even sensually perceptible to our nerves of smell, yet act a peculiar part in the mutual attraction and repulsion of living creatures. Let us, however, consider that already in grey antiquity soul and spirit are distinguished, since the first was distinguished as the principle which animates our bodies and is independent of our will, but the latter designated as the immortal Psyche, which returns to its Creator after the body has ceased to live. Only in later times custom united these two conceptions, which evidently ought to be separated, and to designate the Psyche of the ancients "Soul" in our mother tongue. Prof. Jæger ought to have considered this when making use of this expression for his quite modern theory, for thereby he would have avoided many an offense to every Christian, particularly he would not have drawn upon himself so many unmerited attacks from other quarters, particularly from such people as did not consider it worth their while to reflect over what he had written but in order to pass judgment on him picked out only a few separate quotations from his writings, often enough wrongly reproduced by the newspapers, and appearing paradoxical. We premise these few remarks because when we use the word "soul" in our dissertation upon Neural Analysis we wish to have it understood merely in the sense in which Jæger uses it, and not in that of general colloquial usage, or in that of the tenets of our religion.

According to philosophical conceptions, *Will* is the relation of thinking to acting. By means of our volition we stop actions in one case; in another we direct them. Every action — an activity of motion — represents *the legitimate solution of a tension occurring in our organs of thinking and sensation*, and appears, consequently, in relation to this tension as a solving, delivering, in short, as a critical deed, which is necessarily succeeded by a shorter or longer relaxation (apathy). Now, the central organ of our will is the brain. The brain, composed of ganglia or nerve-cells and of nerve-fibres, is the seat of the mind, for numerous observations made on healthy and sick persons have demonstrated that our sensation and thinking is dependent upon a healthy condition of the brain—nay, even upon certain parts of it. We become conscious of the things outside of us only through changes brought about in these central points of the brain. The way in which such changes are produced is by the organs of the senses. The latter—optical nerve, auricular nerve, etc.—are affected by exterior influences, and the changes obtaining in them are transmitted by simple or complex nerve-connection to the brain. One impression received from without may leave us quiet; another, however, excites our volition, and may be transmuted into motion by the brain. Another way by which our mental apparatus may be influenced is by the blood coursing to the brain. Our mental faculties, dependent upon the brain and the exercise of will flowing from them, are consequently conditional upon relations of tensions in which we either voluntarily place this organ, or in which, without our will, it is placed by the anatomical arrangements of our body and the physiological occurrences connected with it (circulation of blood, etc.).

These relations of tension, now, are not always the same. They are subject to constant change. They depend partly upon the general chemical condition of mixture of the mass of fluids of our body, partly also upon impressions which our organs of sense receive from without and transmit to the brain. Prof. Jæger is to be credited with having made the study of these *relations* of tension the main study of his life, for he is the first who has proved that they may be expressed in mathematical formulæ, since for their measurement he made use of a chronoscope.

This instrument has been used in astronomy for years for the measurement of "personal equation." Comets, planets and planetoids are, as is well known, observed from many observatories simultaneously, and in order to be enabled to observe accurately, the astronomer must determine the time, in fractions of a second, in which the celestial body enters the crossed spider-lines in his equatorial. The ascertained times must coincide with those of astronomers at other observatories, if the observations and the calculations based thereon are to be correct. For the places of observation this time can be pretty accurately calculated. One part of the earth, chiefly Europe, has been very accurately measured, and the distances of the separate observatories from each other are accurately known. As to the astronomer the case is different, for he must mark the entrance of the celestial body into the crossed spider-lines by a pressure upon the key of his Hipp chronoscope; and this or some other instrument constructed according to similar principles is, moreover, connected telegraphically with the astronomical clock, and there also indicates the exact time in fractional divisions of a second; the astronomer has, therefore, touched the key at the moment when he saw the star in the thread-cross. Those who do not know this matter might opine that our astronomer had marked the correct time. But this is by no means the case. For, from the moment when we notice an object with our eye, upon the retina of which the corresponding changes take place, until that moment when our finger, in consequence of the impulse imparted to it from the brain, presses upon the key, there elapses a certain time which we, following Jæger, will call *nerve time.* Prof. Helmholtz, the well known physiologist in Berlin, likewise measured this interval of time several years ago, and ascertained it to amount, on an average, with different people, to from 100 to 150 mill-seconds. Now, although this is a very small interval, yet for astronomical calculation this statement of Helmholtz is not sufficient, for every astronomer knows that this nerve time is subject to a great change, a change amounting with the same operator to between 50 and 300 mill-seconds, i. e., in different nights of observation. As a practical man, the astronomer does not inquire into the causes of these considerable differences, but he is satisfied shortly before beginning his observations to verify his nerve time by measuring it 40 or 50 times successively and deducing the average value. This procedure he calls "personal equation."

Physiologists, studying the functions of the human body, had, therefore, necessarily to face the question: *Whence originate these differences?* To have first attempted an answer to this question, and to have made a great number of experiments concerning it, constitute the great merit of Prof. Jæger. He has published his ideas about all the circumstances to be taken in consideration in connection with it, not only in his writings but also in public assemblies. He has furthermore founded his theory of the "Soul" upon it. Yet, the thoughts pronounced by him were too new. They moved the termini of knowledge another distance outward, and the form in which this was done, on the part of Dr. Jæger, not only led to contradictions of all that he had said and written, but to direct attacks upon his teachings, and even upon his person.

Considering the importance which the experiments instituted by Jæger, have for homœopathy, and in view of the stormy opposition they will presumably excite in hostile quarters, we are obliged, before discussing the experiments themselves, to make some remarks upon Jæger's person; for our readers would not otherwise be enabled to meet the objections raised by anti-homœopathists. Professor Dr. Jæger is an approved physician, but some years since he devoted himself to anthropology and zoology, and his name is honorably mentioned in both these domains of investigation. He discovered a variety of new things and is an active contributor to journals of these branches and an author of works in this line. The considerable fame which he acquired induced the government of Wurttemburg to offer him the professorship of zoology and anthropology at the Royal Polytechnicum, as well as at the Royal Veterinary School. He accepted it and has now been living in Stuttgart for more than ten years, esteemed both as man and *savant.* It is only for the last two years, since he proclaimed his theory of the soul at the congress of naturalists in Baden-Baden, that a peculiar agitation against him has arisen, which, in its excesses, might even be called vulgar and malignant. First, some detached sentences from his discourse delivered in Baden-Baden were arbitrarily dissevered from their context and scattered by the daily press. Then the agitation spread to the scientific journals by the endeavor to undo all that he had ever accomplished, and which had formerly been acknowledged, and by calling him nothing but the "Soul-smeller" and "Man-of-the-odorous-Soul." The indecent tone of the journals was pushed to such extremes that most German papers were not ashamed to reprint the following item from a Wurttemburg newspaper: "The famous Dr. Jæger, of the curious doctrine of Soul-odor, was run over by a locomotive at the station of Nordlingen and lost both his legs." A severe calamity which had happened to some other Dr. Jæger, was thus announced in a jesting tone, almost like a good thing, simply because they supposed it had to do with the "Man-of-the-Soul-odor." The literary journeymen and the mob have taken hold of him for the last two years and everybody believes he can establish his adverse judgment about him by simply shouting "Soul-smeller." No trouble is taken to read accurately what Jæger wrote about the subject, nor is it considered necessary to make a single one of his experiments after him with the same apparatus and under the same conditions. Only in the latter case would there be any justification for contradiction, but never for derision. For even assuming the case that Jæger's doctrines form no complete whole, that they have but an embryotic stamp, still no fair-minded man will dare to mock an investigator who tries to contribute to the solution of the greatest enigma, represented by man, not in the speculative, philosophical way, but in that of the exact mathematic-physical experiment.

The manipulation of the neural-analytical apparatus pre sup-poses a certain practice. The object of optical perception with the astronomer is a star, at the appearance of which, in a certain part of its orbit, he exercises a pressure upon the key; whilst in the neural-analytical experiments of Prof. Jæger, the beginning of the movements of the hands at the upper dial does duty for an optical signal. The key must therefore be cautiously pressed, and the pressure be at once stopped upon the movement of the index. With some energy and attention, however, this difficulty is overcome, and finally one works the apparatus so mechanically and in such equal intervals, that he obtains always the same or quite similar numbers in perfect mental and bodily rest, undisturbed by exterior and interior influences. Only after having thus obtained such familiarity with the instrument, exact neural investigations in the meaning of Jæger can be proceeded with, and—as cannot be sufficiently emphasized—an objective judgment about the value or worthlessness of this new proceeding is not possible before that stage.

In order to measure your own nerve-time, you sit down before the apparatus, note the position of both indices, place your left hand upon the key, set the clock-work going with your right hand, look with the greatest attention at the yet motionless hands and slowly press the key. At the moment when the current is completed, the hands rotate and in the same moment in which this is perceived, you quickly relinquish the pressure of your left hand upon the key, whereupon the index stops. The division upon which it stopped, is read off and noted down, and the difference between the first-noted and the present position of the hands indicates the time which elapsed from the moment when we saw the hand moving to the one in which, by removing the finger of our left hand, we caused it to stand still; that is the *nerve-time.* This nerve-time is a something, only partially dependent upon the degree of intensity of our attention and our will, for although, by *willing* it energetically, we can shorten this time somewhat, yet each of the inward and outward impressions to be mentioned hereafter, may retard or prolong it; so that we must admit that the condition of the transmitting capacity of our nervous system has nothing to do with our will. For it occurs that, when a person intimate with the apparatus investigates substances which extraordinarily heighten his nervous susceptibility, the nerve-times become, not only very short and amount to but a few mill-seconds, but that the hand does not get into motion at all, so that only the noise arising in the clock by the attraction of the axis of the hand is heard and we sit apparently powerless before the instrument and often obtain again shorter nerve-times only after ten to twenty acts. And conversely certain impressions may considerably prolong our nerve-time.

The nerve-time is then, in the manner described above, measured at regular intervals of ten to twenty seconds, about a hundred times successively and the progress or the motion of the hands noted each time; for instance:

| | | |
|---|---|---|
| 60 mill-seconds | —— | difference, 60 mill-seconds. |
| 125 " " | —— | " 65 " " |
| 175 " " | —— | " 50 " " |
| 225 " " | —— | " 50 " " |
| 275 " " | —— | " 50 " " |
| 325 " " | —— | " 50 " " |
| 385 " " | —— | " 60 " " |
| 440 " " | —— | " 55 " " |
| 495 " " | —— | " 55 " " |
| 555 " " | —— | " 60 " " |
| —— | | —— |
| 555 mill-seconds | —— | " 555 mill-seconds. |

The average value of these ten figures amounts therefore to 55 mill-seconds; the divergences of the separate figures, 60, 65, 50, etc., marked upon a strip of paper ruled in square millimetres, seriatim in regular distances by points, distant from the upper ground-line as many quarter-millimetres as the nerve-time amounted to mill-seconds, and these points continuously connected with their neighbors by lines, gives a *detail-curve*. If the nerve-times at susceptible conditions of the nervous system are very short ones, the points are placed not far from the upper ground-line, the lines uniting them in zig-zag way run close to the edge of it, whilst with a lower energy, corresponding with the greater numerical values, they approach the lower ground-line and thus furnish a perspicuous image of the condition of our nervous system—for they present to us the change to which it is subject, in diagrams. By adding the average values of ten of these decades (constituting 100 acts) and dividing by ten, we obtain a decade average. To the latter we will return later on when discussing the numerical values for homœopathic medicinal potencies as furnished by Prof. Jæger.

The constitution of the detail-curve depends upon the total chemical condition of the mass of mixed fluids of our body, and everything that changes it produces also changes in the configuration of the curve; all substances that we eat, drink and breathe; the changes taking place in the digestive process, finally also all mental disturbances. But of quite particular interest for us, are the curves resulting from the *inhaling* of certain substances and the average values of them, for upon that the neural-analytical examination of homœopathic medicinal potencies is founded.* Most of our readers might presume from the beginning, that these, in their higher dilutions, have no other smell than that of the alcohol with which they are prepared; and yet the detail-curve shows a very considerable difference between the two. This is probably owing to our organ of smell, the nose, through which at normal inhalation the respired air passes, being with most people not very sensitive, or at least not making us directly conscious of certain things. Are there not certain asphyxating gases that we do not smell at all, whilst, when inhaled, they poison our blood and we become first aware of them by the incurred symptoms of disease? In a similar manner the changes of the neural-analytical curves at the inhaling of potencies or other things which according to Jæger influence our nervous system characteristically, might be explained; taken up by the blood in the lungs they thereby influence the quickness of the transmitting capacity of our nerves—the *nerve-time.*

To obtain exact results, there is required, as with every other test of medicines, an extremely careful mode of living, to which Prof. Jæger has devoted a separate chapter in his recently published book.

After you have now, as directed above, measured 100 acts in perfect rest, you pour a small quantity of the same alcohol with which the homœopathic potency to be examined has been prepared, into a

---

* Prof. Jæger, moreover, found a great similarity to exist between the inhaled and the swallowed substance in the formation of the curves. He calls those obtained by swallowing, *Genogram*, those by inhaling, *Osmogram.* He gives the preference to the latter method since the inhaling of a substance can be intermitted at pleasure.

small glass vessel, and set the apparatus going again while at the same time inhaling the alcohol. The curve now obtained shows a very essential difference from that obtained in rest, even with persons that treat the apparatus as mere tyros. In spite of all trouble taken in spite of our strongest volition, it is not possible for any one to bring the nerve-times again into the normal balance. With some persons they are considerably lengthened, with others they are shortened, or they move (in the Osmogram) in a confused zig-zag up and down.

(TO BE CONCLUDED NEXT ISSUE.)

